# Galectin-9 blockade synergizes with ATM inhibition to induce potent anti-tumor immunity

**DOI:** 10.7150/ijbs.79852

**Published:** 2023-01-16

**Authors:** Shuang Zheng, Jiaming Song, Dongli Linghu, Riyao Yang, Boning Liu, Zhen Xue, Qihui Chen, Chengjie Liu, Diansheng Zhong, Mien-Chie Hung, Linlin Sun

**Affiliations:** 1 Tianjin Key Laboratory of Lung Cancer Metastasis and Tumor Microenvironment, Lung Cancer Institute, Tianjin Medical University General Hospital, Tianjin 300052, P. R. China.; 2 Department of Medical Oncology, Tianjin Medical University General Hospital, Tianjin 300052, P. R. China.; 3 Department of Lung Cancer Surgery, Tianjin Medical University General Hospital, Tianjin 300052, P.R. China.; 4 Antibody Therapeutics, Inc., Hayward, CA, USA.; 5 Department of Molecular and Cellular Oncology, The University of Texas M. D. Anderson Cancer Center, Houston, Texas 77030, USA.; 6 Graduate Institute of Biomedical Sciences and Center for Molecular Medicine, China Medical University, Taichung 40402, Taiwan.

**Keywords:** Cancer immunotherapy, DNA damage response, cGAS-STING, galectin

## Abstract

Although current cancer immunotherapies that target PD-1/PD-L1 immune checkpoint to reinvigorate exhausted T cells have achieved impressive clinical outcomes, only a small proportion of patients respond. New therapeutic targets are therefore needed to be identified to further unleash the anti-tumor potential of T cells and benefit more patients. Galectin-9 (Gal-9), initially identified as a ligand for TIM-3 to induce T cell death, acts as an immunosuppressive regulator in the tumor microenvironment (TME) but its potential as a therapeutic target remains largely elusive. Here we show that antibody neutralization of Gal-9, in combination with inhibition of Ataxia telangiectasia mutated (ATM), a kinase essential for DNA damage response (DDR), is a promising modality for cancer immunotherapy. Genetic depletion of ATM in tumors markedly potentiated anti-Gal-9 therapy in a syngeneic mouse model. Mechanistically, ATM inhibition greatly upregulated Gal-9 expression and secretion in a variety of human and murine tumor cells via the cGAS-STING-interferon β (IFNβ) innate immune pathway. Combination of Gal-9 inhibition with AZD1390, a selective ATM inhibitor currently evaluated in clinical trials, significantly suppressed tumor growth and prolonged survival in multiple syngeneic mouse models, including the poorly-immunogenic LLC lung tumors that do not respond to PD-1/PD-L1 blockade, concomitant with increased T cell infiltration. These results reveal Gal-9 induction via STING/IFNβ signaling as an important mechanism mediating tumor immune escape that could be targeted for cancer immunotherapies, and unveil a novel anti-Gal-9-based combination strategy for cancer immunotherapies in a wide variety of malignancies, including those resistant to PD-1/PD-L1 blockade.

## Introduction

Therapeutic blockade of the programmed cell death protein 1 (PD-1) and the cytotoxic T-lymphocyte associated protein 4 (CTLA-4) immune checkpoints has emerged as a powerful strategy for cancer treatment in multiple cancer types by reinvigorating exhausted T cells 1,2. However, the remarkable responses are currently limited to a minority of patients, highlighting the need for exploring new therapeutic targets to further unleash the anti-tumor potential of T cells and benefit more patients. Galectin-9 (Gal-9), a member of the galectin family, was initially identified as a ligand for TIM-3 to induce T cell death 3. Elevated expression of Gal-9 is associated with a more terminally exhausted T cell phenotype 4. Gal-9 protein has two conserved distinct carbohydrate-recognition domains (CRD) for binding β-galactosides, capable of crosslinking glycoproteins and modulating cell signaling 5. Gal-9 primarily facilitates immunosuppression in the tumor microenvironment (TME) by interacting with multiple cell surface receptors on immune cells, such as TIM-3, Dectin-1 and CD44 3,6,7. It is constitutively expressed in antigen-presenting cells (APCs), and can be induced in tumor cells by interferon (IFN) β and γ 8. The expression of Gal-9 is aberrantly elevated in multiple cancer types and largely correlates with poor patient survival 9-12. However, the regulation of Gal-9 in the context of cancer therapies and its potential as a therapeutic target remain largely elusive.

The defect in DNA repair is a hallmark of cancer. DNA damage response (DDR) pathway is essential for the maintenance of genomic integrity 13,14. The deficiency of DDR has been shown to be a critical contribution to tumor immunogenicity. Mounting evidence supports that DDR-targeted therapies can induce innate immunity and promote antitumor immune response 15. Ataxia telangiectasia mutated (ATM) is an important DDR kinase which senses and mediates the repair of DNA double-strand breaks (DSBs) by homologous recombination (HR) 16,17. Clinically, hereditary mutations of ATM result in ataxia telangiectasia (A-T), a syndrome characterized with neurodegeneration, immunodeficiency, radiation sensitivity and cancer predisposition 18. Somatic mutations or deletions of ATM are commonly found in a variety of cancers 19. Inhibition of ATM results in reduced HR and compromised DSB repair, leading to cell cycle arrest or cell death. ATM inhibitors have been extensively evaluated in clinical trials 20 and found to sensitize tumors to chemotherapy, radiation, and other DDR inhibitors in multiple cancer types 19. Moreover, prior study indicates that ATM deficiency activates cGAS/STING through promoting cytoplasmic leakage of mitochondrial DNA, and subsequently induces type I interferon (T1IFN)-mediated innate immune response in certain cancer types 21,22. Although ATM inhibition has been shown to potentiate anti-PD-1 therapy 22, the therapeutic efficacy of their combination is moderate, suggesting that there might be additional unknown mechanisms that mediate immune escape. Better understanding of the immunological effect of ATM inhibition on the TME will help to identify new targets to enhance its efficacy.

In this study, we demonstrate that Gal-9 is induced by ATM inhibition through cGAS-STING-IFNβ pathway in a variety of tumor cells. Importantly, Gal-9 blockade combined with genetic depletion or pharmaceutical inhibition of ATM synergistically induces potent anti-tumor immunity and markedly reduces tumor growth in syngeneic mouse models. These findings highlight Gal-9 as a promising therapeutic target in ATM-silenced tumors, and provide a rationale for combining ATM inhibition with anti-Gal-9 as novel strategy for cancer immunotherapy.

## Materials and Methods

### Cell lines and reagents

All cell lines were obtained from the American Type Culture Collection (ATCC, Manassas, VA, USA), independently validated by short tandem repeat (STR) DNA fingerprinting, and tested negative for mycoplasma infection. NCI-H157, A549, NCI-H1299, HeLa, BT549, MDA-MB-231, CT26, B16-F10, MC-38, LLC and 293T cells were cultured in RPMI 1640 or DMEM media, supplemented with 10% fetal bovine serum (Thermo Fisher Scientific, Waltham, MA, USA) at 37 °C in a humidified atmosphere with 5% CO_2_. Inhibitors for ATM (KU-60019, AZD1390, AZD0156), STING (H151), p-TBK1 (GSK8612), p-STAT1 (fludarabine) were purchased from Selleck (Houston, TX, USA). Human IFNβ and Galectin-9 ELISA assay Kits were purchased from R&D SYSTEMS (Minneapolis, MN, USA). Anti-human Gal-9 antibody was purchased from BioRad (Hercules, CA, USA). Anti-mouse Gal-9 antibody and IgG isotype control were purchased from BioXCell (Lebanon, NH, USA). Antibodies against cGAS, STING, p-STING(Ser366), p-TKB1(Ser172), TKB1, p-STAT1(Tyr701), STAT1 and Actin were purchased from Cell Signaling Technology (Cambridge, MA, USA). Anti-mouse fluorochrome-conjugated antibodies including FITC-CD4, Percp/Cy5.5-CD8, AF700-CD3, APC/Cy7-CD45, PE/Cy7-IFNγ antibodies were purchased from BioLegend (San Diego, CA, USA).

### Real-time PCR

Real-time PCR analysis was performed as described previously 23. In brief, total RNA was isolated using Trizol reagent (Invitrogen, Carlsbad, CA, USA). cDNA was prepared using HiScript III 1st Strand cDNA Synthesis Kit (Vazyme, Nanjing, China) with 1 μg of total RNA according to the manufacture's protocol. Real-time PCR reactions were then performed in a mixture containing 1×SYBR Green Mix (Vazyme, Nanjing, China), specific primers and cDNA template using the Applied Biosystem 7900 system (USA). The relative expression levels of target genes were normalized to that of GAPDH. Primers used for different target genes are listed in Supplemental Table 1.

### Immunoblot analysis

Immunoblot analysis was performed as described previously 24. In brief, proteins were resolved by SDS-PAGE and transferred onto polyvinylidene difluoride (PVDF) membranes (Millipore, Burlington, MA, USA). The membranes were blocked in Tris-buffered saline containing 0.2% Tween 20 and 5% fat-free dry milk, and then incubated with primary antibodies at 4 °C overnight, and horseradish peroxidase-conjugated secondary antibodies at room temperature for 1 hour. After washes, specific protein bands were visualized using chemiluminescence detection reagent (Millipore, Billerica, MA, USA) according to the manufacturer's instructions (Pierce Biotechnology, Waltham, MA, USA).

### ELISA

ELISA analysis was performed as described previously 8. Briefly, Nunc Maxisorp ELISA Plates (BioLegend) were coated with capture antibody in PBS at 4 °C overnight according to the manufacture's protocol (R&D SYSTEMS, Minneapolis, MN, USA). The coated plates were then washed, blocked with BSA and incubated with conditional media. This was followed by incubation first with the detection antibody and then with HRP-labeled IgG antibodies. After washes, the plates were incubated with TMB substrate solution for 20 mins, and the reaction was terminated by the addition of H_2_SO_4_. Absorbances at 450 nm with a reference wavelength of 570 nm was then measured using a microplate reader (SpectraMax M5, Molecular Devices, CA, USA).

### Crystal violet staining

Cells were seeded in appropriate dilutions to form colonies in 1-3 weeks. Colonies were fixed with 4% paraformaldehyde, stained with 0.5% crystal violet staining solution at room temperature for 30 min, and then washed three times with deionized water.

### Cell proliferation assay

Cell proliferation was determined using Cell Counting Kit-8 (CCK8) assay (Bimake.cn, Shanghai, China). Cells were seeded in 96-well plates to grow for 2-4 days, and then incubated with CCK8 reagent at 37 °C for 1-3 hours. The absorbance at 450 nm was measured with a microplate reader (SpectraMax M5, Molecular Devices, CA, USA).

### Generation of stable knockdown cells by lentiviral transduction

The plasmids encoding shRNA targeting human STING, IFNAR1 or murine ATM were obtained from Shanghai Genechem Co., Ltd. n. The sequences for different shRNAs are listed in Supplemental Table 2. To generate stable knockdown cells, 293T cells were co-transfected with 4 μg shRNA expressing construct, 3 μg pCMV-dR8.2 and 1 μg pCMV-VSVG helper construct using Lipofectamine 3000 reagent (Life Technologies, Carlsbad, CA, USA). The virus stocks were harvested from the culture medium after 2 days and then filtered to remove non-adherent 293T cells. To select the cells that stably express the specific shRNA, cells were plated at subconfluent densities and transduced with a cocktail containing 1 ml virus stock, 1 ml regular medium and 8 μg/ml polybrene, and then selected with 2 μg/ml of puromycin 48-72 hours after lentiviral transduction.

### Analysis of TILs by flow cytometry

Approximately 5×10^5^ CT26 cells were inoculated subcutaneously into the left flank of BALB/c mice and treated with isotype control, AZD1390, anti-Gal-9 antibody (BioXCell, Lebanon, NH, USA), or the combination AZD1390 and anti-Gal-9 antibody. Tumors were excised on day 14 after tumor inoculation, and then mechanically minced and incubated in DNase I (50ug/mL, Solarbio) and collagenase IV (3 mg/mL, Solarbio) for 20 minutes at 37 °C. The dissociated cells were then passed through a 70 μm cell strainer (BD). The filtered cells were stained with Zombie Violet™ Fixable Viability Kit (BioLegend), blocked with FcR Blocking Reagent (Miltenyi), and stained with the indicated surface antibodies in staining buffer (PBS containing 1% BSA and 0.1% NaN3) for 20 minutes on ice. Intracellular antibodies were added after fixation and permeabilization according to the manufacturer's instructions (Thermo Fisher Scientific). The stained cells were analyzed by Flow Cytometer (BD Biosciences, CA, USA).

### Animal models

All experiments were performed in accordance with the guidelines for the Care and Use of Laboratory Animals of Tianjin Medical University Hospital (Tianjin, P.R. China). 6-8-week-old male C57BL/6J mice and female BALB/c mice were purchased from Beijing Vital River Laboratory Animal Technology Co. Ltd and housed in laminar flow cabinets under specific pathogen-free conditions. For CT26 subcutaneous model, a total of 5×10^5^ CT26 cells were injected subcutaneously into left flank of the BALB/c mice. For MC-38 subcutaneous model, a total of 5×10^5^ MC-38 cells were injected subcutaneously into left flank of C57BL/6J mice. For LLC subcutaneous model, a total of 5×10^5^ LLC cells were injected subcutaneously into left flank of C57BL/6J mice. After tumors were palpable, mice were randomized into treatment cohorts as isotype control, AZD1390 (5 mg/kg for 9 times), anti-Gal-9 antibody (100 μg/dose for 3 times) (BioXCell, Lebanon, NH, USA), or the combination AZD1390 and anti-Gal-9 antibody. Tumor size and body weight were measured every 2-3 days. Tumor volumes were calculated using the formula: ab^2^/2 (a: length, b: width). According to animal ethics, the mice were sacrificed when tumors reached 2000 mm^3^ in size.

For ATM-silenced CT26 subcutaneous model, a total of 1×10^6^ vector control or ATM KD CT26 cells were injected subcutaneously into right flank of the BALB/c mice (female, 6-8 weeks old). After the tumors were palpable, mice were randomized and treated with anti-Gal-9 (100 μg/dose) or IgG isotype control (BioXCell, Lebanon, NH, USA) intraperitoneally every 3 days for 3 times. Tumor size and body weight were measured every 2-3 days. Tumor volumes were calculated using the formula: ab^2^/2 (a: length, b: width). According to animal ethics, the mice were sacrificed when tumors reached 2000 mm^3^ in size.

### Statistical analysis

Statistical analysis was performed using the GraphPad Prism software. All data are presented as mean ± standard deviation (S.D.). Unpaired two-tailed t tests or one-way ANOVA were used for comparison between two groups. P values < 0.05 were considered to be statistically significant.

## Results

### Genetic depletion of ATM sensitizes tumors to anti-Gal-9 therapy

**Figure 1:**
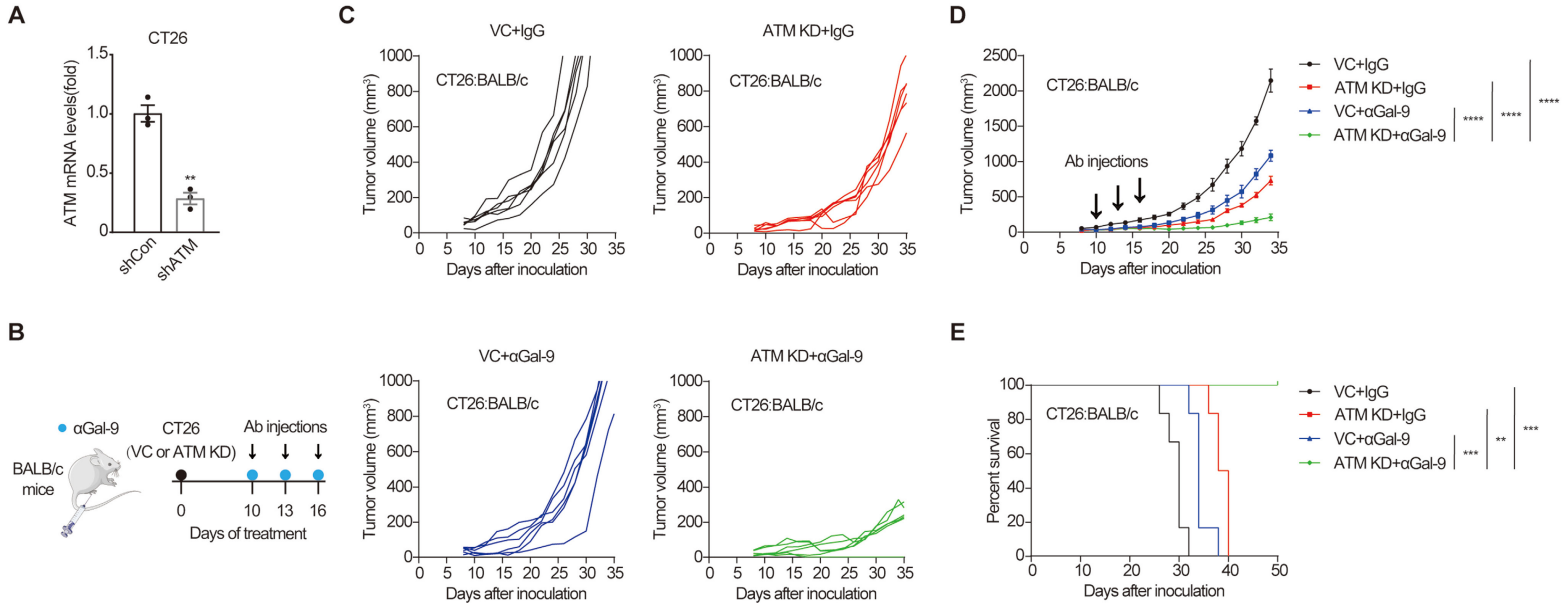
** ATM depletion sensitizes tumors to anti-Gal-9 therapy in CT26 syngeneic mouse model. A.** RT-qPCR analysis of ATM mRNA in vector control and ATM-KD CT26.** B.** A diagram of treatment strategy. BALB/c mice were inoculated with vector control (VC) or ATM-KD CT26 cells and then treated with isotype or anti-Gal-9 antibody (100μg /dose for 3 times).** C.** Tumor growth curves of individual BALB/c mice inoculated with VC or ATM-KD CT26 cells and subjected to indicated treatment (n = 6 mice/group). **D.** Average tumor growth of mice inoculated with VC or ATM-KD CT26 cells and subjected to indicated treatments. Error bars represent SEM of the means. Anti-Gal-9 antibody treatment schedule is indicated by arrows. **E.** Survival curves of BALB/c mice inoculated with VC or ATM-KD CT26 cells and subjected to the indicated treatments (n = 6 mice/group). n.s., not significant; *, P < 0.05; **, P < 0.01; ***, P < 0.001.

Analysis of ATM mutation frequency in The Cancer Genome Atlas (TCGA) cohort using c-Bioportal 25,26 indicated that ATM is frequently mutated in multiple human cancers, with a higher mutation frequency of 40% in mantle cell lymphoma, and approximately 20-35% in cutaneous, uterine, lung and colorectal cancers (Fig. S1A). To examine the efficacy of anti-Gal-9 therapy in ATM-silenced tumors, we first established an ATM knockdown (KD) cancer cell line by transducing the murine CT26 colon cancer cells with lentiviruses encoding shRNA for ATM. Substantially reduced ATM expression was verified using real-time PCR (Fig. 1A). ATM depletion did not affect the growth of tumor cells *in vitro*, as demonstrated by crystal violet staining and CCK-8 assay (Fig. S1B and C), suggesting that there were no cell-intrinsic growth defects in ATM KD cells. We then inoculated syngeneic BALB/c mice with control or ATM KD CT26 tumor cells, and found that ATM-depleted cells formed smaller tumors compared with vector control (VC) (Fig. S1D), indicating a tumor-suppressive effect of ATM depletion. Interestingly, while anti-Gal9 (RG-1) treatment (100 μg/dose for 3 times) partially suppressed the growth of control tumors, it resulted in almost complete growth suppression in ATM-KD tumors, correlated with markedly prolonged mouse survival (Fig. 1B-E), suggesting a remarkable synergy between Gal-9 inhibition and ATM depletion. Together, these results indicated that genetic depletion of ATM significantly potentiates anti-Gal-9 therapy in a syngeneic mouse model.

### ATM inhibition induces Gal-9 expression and secretion in cancer cells

**Figure 2:**
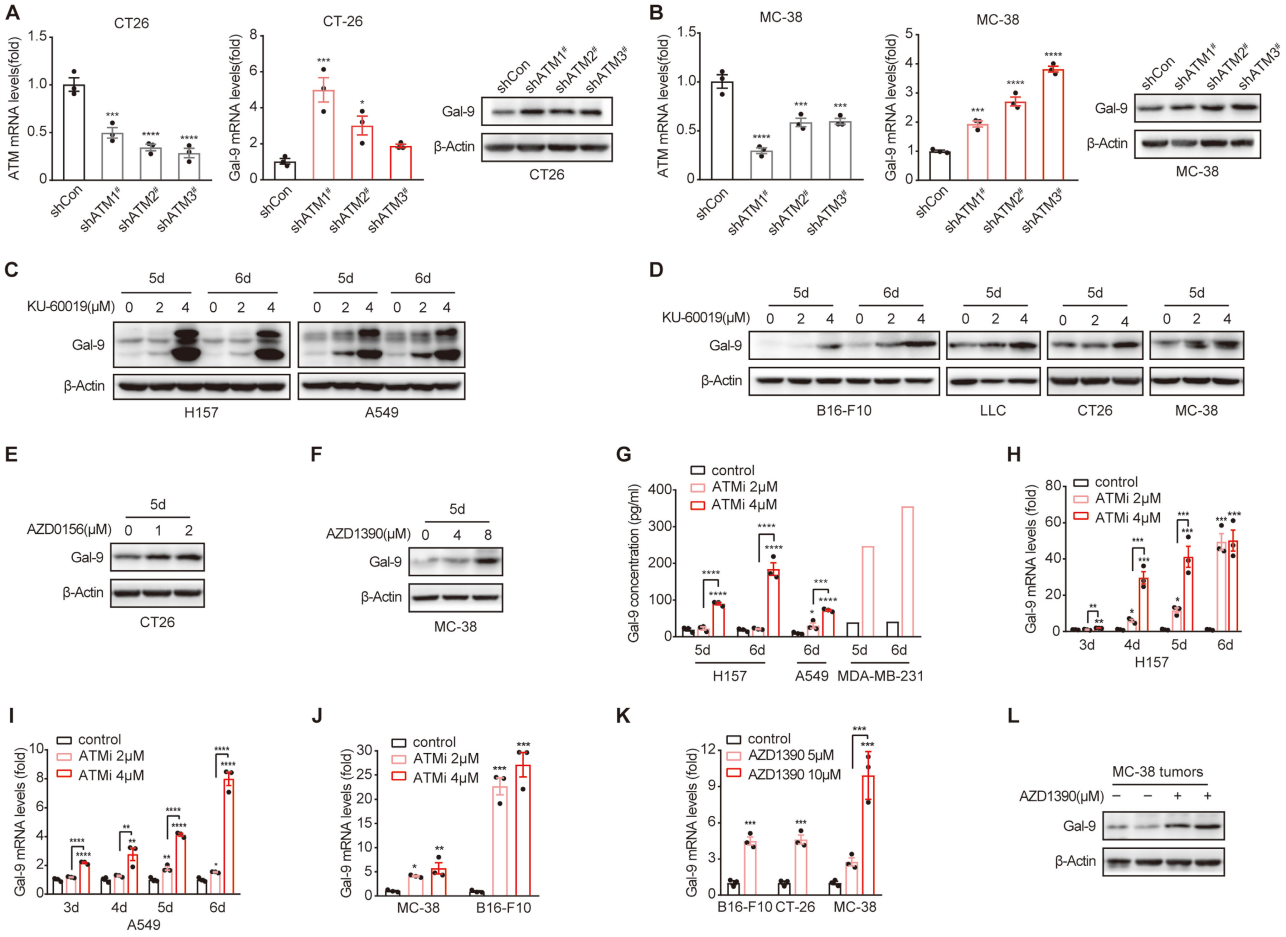
** ATM KD or inhibition results in increased Gal-9 expression and secretion in a variety of tumor cells. A**. RT-qPCR and immunoblot analysis of ATM and Gal-9 in vector control and ATM-KD CT26. **B.** RT-qPCR and immunoblot analysis of ATM and Gal-9 in vector control and ATM-KD MC-38 cells. **C-F.** Immunoblot analysis of Gal-9 in H157, A549, B16-F10, LLC, CT26 and MC-38 cells treated with KU-60019 (2 μmol/L or 4 μmol/L), AZD0516 (1 μmol/L or 2 μmol/L), AZD1390 (4 μmol/L or 8 μmol/L) or DMSO (vehicle control) for indicated time. **G.** ELISA analysis of Gal-9 in H157, A549 and MDA-MB-231 cancer cells treated with KU-60019 (2 μmol/L or 4 μmol/L) or DMSO (vehicle control) for indicated time. **H and I.** RT-qPCR analysis of Gal-9 mRNA levels in H157 and A549 cancer cells in response to KU-60019 (2 μmol/L or 4 μmol/L) or DMSO (vehicle control) for indicated time. **J.** RT-qPCR analysis of Gal-9 mRNA in MC-38 and B16-F10 cells in response to KU-60019 (2 μmol/L or 4 μmol/L) or DMSO (vehicle control) for 6 days. **K.** RT-qPCR analysis of Gal-9 mRNA in MC-38, B16-F10 and CT26 cells in response to AZD1390 (5 μmol/L or 10 μmol/L) or DMSO (vehicle control) for 6 days. **L.** Immunoblot analysis of Gal-9 in AZD1390-treated MC38 tumors harvested from C57BL/6J syngeneic mice. n.s., not significant; *, P < 0.05; **, P < 0.01; ***, P < 0.001.

Next, we sought to investigate the molecular mechanisms underlying the potent efficacy of anti-Gal-9 in ATM-depleted tumors. Given that high levels of intratumoral Gal-9 in the TME is supposed to predict the response to anti-Gal-9 therapy, we hypothesized that ATM depletion might result in Gal-9 induction and thus render these tumors highly sensitive to anti-Gal-9 therapy. To test the possibility, we evaluated the expression of Gal-9 in VC and ATM-KD CT26 cells by RT-PCR and western blot. Indeed, we found Gal-9 mRNA and protein levels were increased in ATM KD CT26 cells compared with those of VC (Fig. 2A). Similar results were found in ATM-silenced MC-38 cells (Fig. 2B). These results suggested that genetic inhibition of ATM leads to Gal-9 induction in tumor cells. To further validate our findings, we treated a panel of human and murine cancer cell lines with a potent and selective ATM kinase inhibitor, Ku-60019, and analyzed Gal-9 protein levels by western blot. Similar to ATM KD, we found that Gal-9 levels were substantially enhanced in response to ATM inhibition (ATMi) in H157 and A549 human non-small cell lung cancer (NSCLC) cells, MDA-MB-231 human triple-negative breast cancer (TNBC) cells, B16-F10 murine melanoma cells, LLC murine lung tumor cells, CT26 and MC-38 murine colon tumor cells (Fig. 2C-D, Fig. S2A). Consistently, Gal-9 was also upregulated in response to AZD0156 and AZD1390, two another ATM inhibitors that have been evaluated in clinical trials (Fig. 2E-F). Given that Gal-9 is a secretory protein, we subsequently evaluated the secretion levels of Gal-9 by ELISA. In agreement, ATM inhibition resulted in a significant increase in the secretion of Gal-9 in H157, A549 and MDA-MB-231 cells (Fig. 2G). To investigate whether ATMi-mediated Gal-9 upregulation occurs at the transcriptional level, we evaluated Gal-9 mRNA levels with or without ATMi treatment. Consistently, Ku-60019 caused an increase in Gal-9 mRNA levels in multiple cancer cells (H157, A549, B16-F10 and MC-38), in a time and dose-dependent manner; Notably, Gal-9 mRNA levels were remarkably elevated on day 6 of Ku-60019 treatment (Fig. 2H-J), which, in comparison, only resulted in slightly upregulated PD-L1 mRNA levels (Fig. S2B). Similarly, AZD1390 treatment also resulted in an increase in Gal-9 mRNA in murine B16-F10, MC38 and CT26 cells (Fig. 2K), further suggesting that ATMi treatment promotes Gal-9 transcription. In addition, Gal-9 expression was found to be increased in ATMi-treated MC38 tumors harvested from C57BL/6J syngeneic mice (Fig. 2L, Fig. S2C). These results indicated that inhibition of ATM results in Gal-9 induction in tumor cells both *in vitro* and *in vivo*.

### ATM inhibition activates cGAS-STING-IFNβ signaling pathway in multiple cancer types

**Figure 3:**
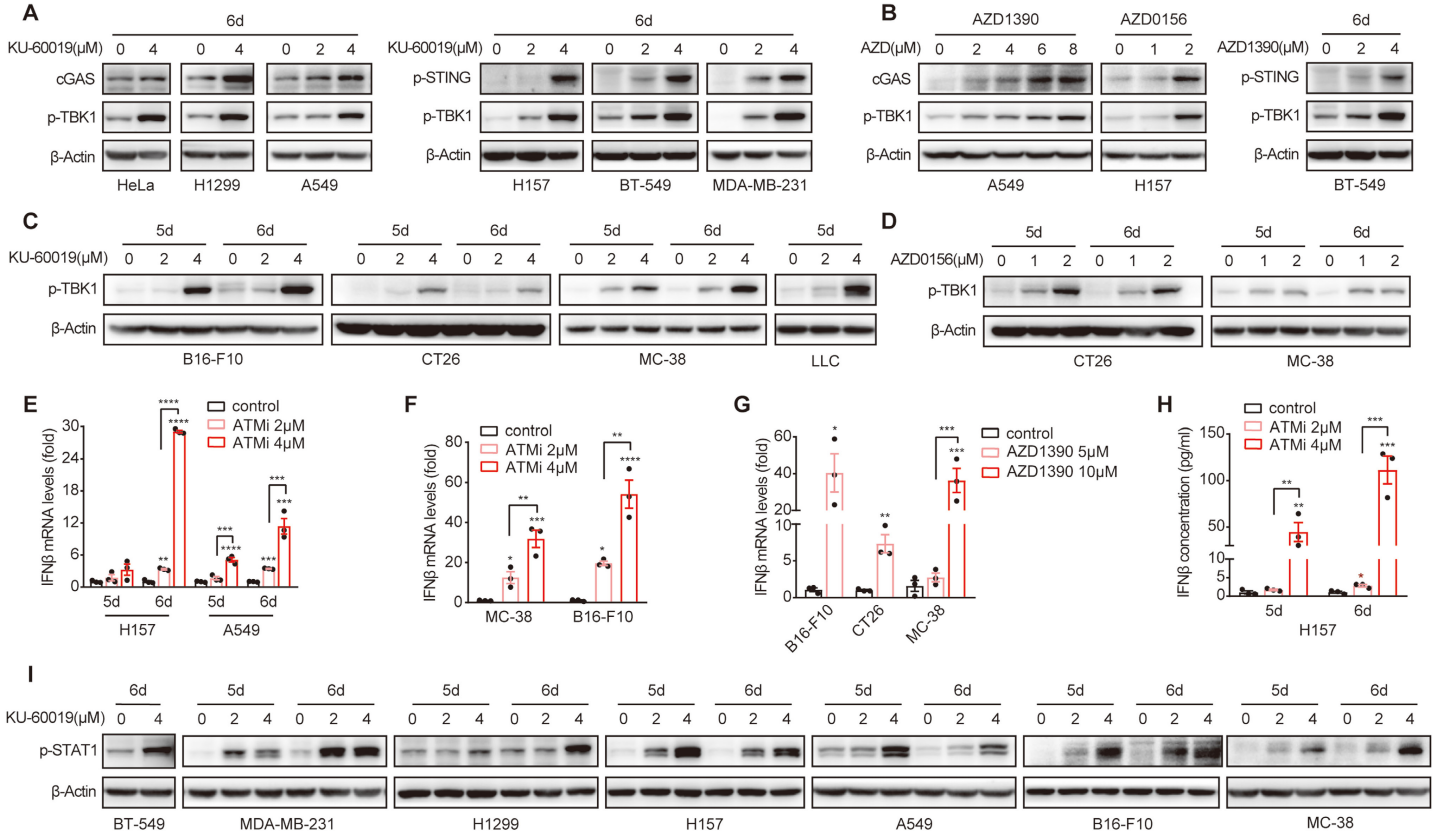
** ATM inhibition activates cGAS-STING-IFNβ signaling in multiple cancer types. A and B.** Immunoblot analysis of cGAS, p-STING and p-TBK1 levels in HeLa, H1299, A549, H157, BT549 and MDA-MB-231 cells in response to KU-60019, AZD1390, AZD0516 or DMSO (vehicle control) for 6 days.** C and D.** Immunoblot analysis of p-TBK1 levels in B16-F10, CT26, MC-38 and LLC cells in response to KU-60019 (2 μmol/L or 4 μmol/L), AZD0156 (1 μmol/L or 2 μmol/L) or DMSO (vehicle control) for 6 days.** E.** RT-qPCR analysis of IFNβ mRNA in H157 and A549 cancer cells in response to KU-60019 (2 μmol/L or 4 μmol/L) or DMSO (vehicle control) for indicated time. **F and G.** RT-qPCR analysis of IFNβ mRNA in in MC-38, B16-F10 and CT26 cells in response to KU-60019 (2 μmol/L or 4 μmol/L), AZD1390 (5 μmol/L or 10 μmol/L) or DMSO (vehicle control) for 6 days. **H.** ELISA analysis of IFNβ in H157 cancer cells treated with KU-60019 (2 μmol/L or 4 μmol/L) or DMSO (vehicle control) for indicated time. **I.** Immunoblot analysis of p-STAT1 levels in BT549, MDA-MB-231, H1299, A549, H157, B16-F10 and MC-38 cells in response to KU-60019 (2 μmol/L or 4 μmol/L) or DMSO (vehicle control) for indicated time. n.s., not significant; *, P < 0.05; **, P < 0.01; ***, P < 0.001.

Next, we investigated the signaling pathway that could mediate ATMi-induced Gal-9 expression. Given the important role of IFNβ in upregulating Gal-9 expression 8, we thus evaluated whether STING-IFNβ pathway is activated in response to ATM inhibition in these cancer cell lines. Indeed, we found that inhibition of ATM led to a robust increase in the expression of cGAS, the phosphorylation of STING and TBK1 in a variety of human cancer cell lines, including HeLa, H157, H1299, A549, MDA-MB-231 and BT549 (Fig. 3A, Fig. S3A and B). Similar results were observed by treating cells with two another ATM inhibitors, AZD1390 and AZD0156 (Fig. 3B). Increased p-TBK1 levels was also found in various murine tumor cells including B16-F10, MC-38 and CT26 and LLC in response to ATM inhibition (Fig. 3C and D). In addition, the expression and secretion of IFNβ was elevated in response to ATM inhibition in various cancer cell lines (H157, A549, MC-38, CT26, and B16-F10) (Fig. 3E-H). Furthermore, the phosphorylation of STAT1, a key transcription factor downstream of IFNβ pathway that contributes to the activation of IFN-stimulated genes, was also enhanced upon ATM inhibition in a panel of human and murine cancer cells (BT549, MDA-MB-231, H1299, H157, A549, B16-F10 and MC-38) (Fig. 3I, Fig. S3C). Together, these results demonstrated that ATM inhibition leads to cGAS-STING activation and IFNβ signaling in multiple cancer types.

### Gal-9 induction by ATM inhibition is mediated by STING-IFNβ pathway

**Figure 4:**
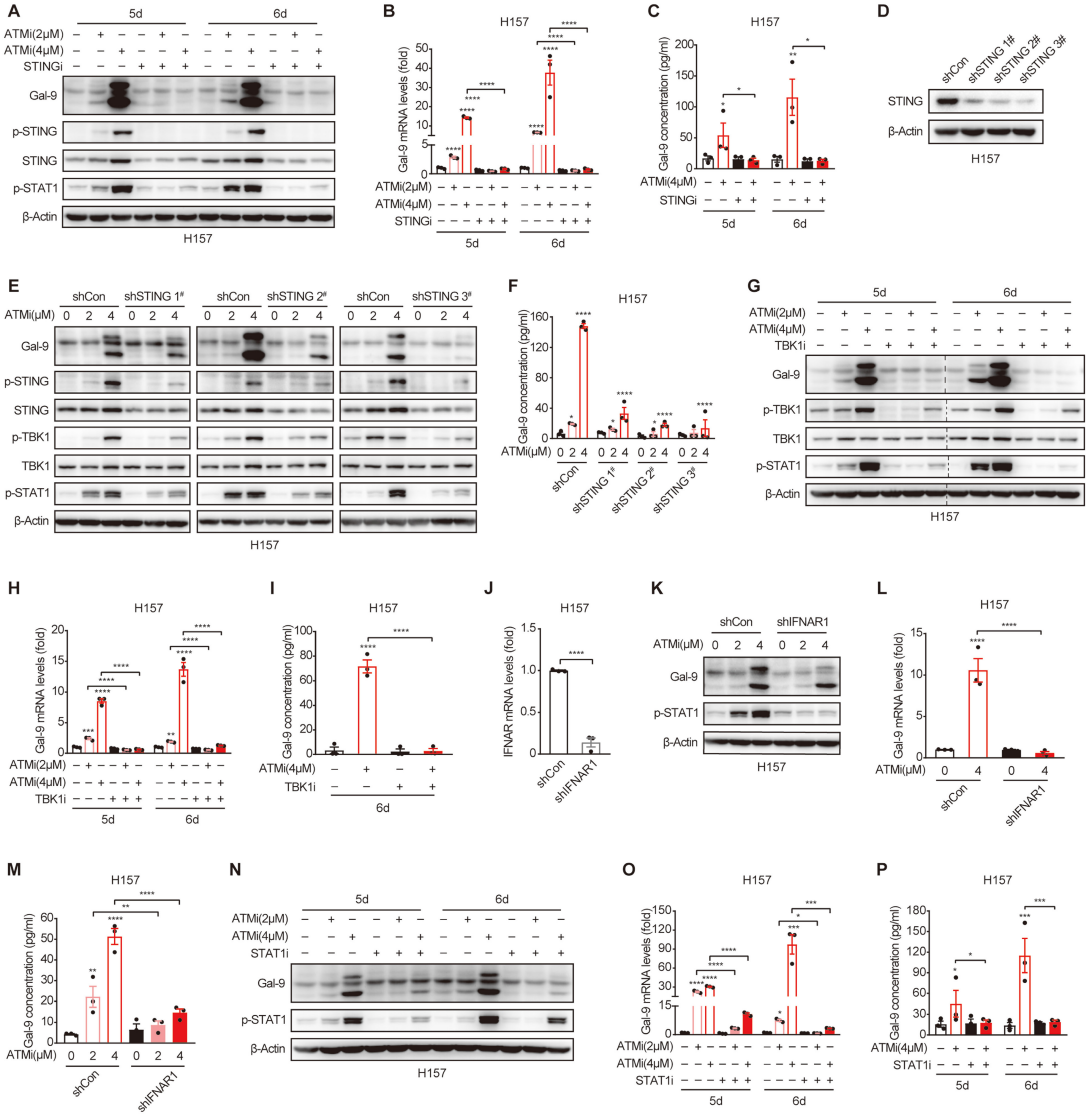
** Gal-9 induction by ATM inhibition is mediated by STING-IFNβ pathway. A-C.** Immunoblot, RT-qPCR and ELISA analysis of Gal-9 in H157 cells concurrently treated with KU-60019 (2 μmol/L or 4 μmol/L) and STING inhibitor (H151, 10 μmol/L) for indicated time. **D.** Immunoblot analysis of Gal-9 in vector control and STING-KD H157 cells expressing three different STING shRNAs. **E-F**. Immunoblot and ELISA analysis of Gal-9 in vector control and STING-KD H157 cells treated with KU-60019 (2 μmol/L or 4 μmol/L) or DMSO (vehicle control) for 5 days.** G-I.** Immunoblot, RT-qPCR and ELISA analysis of Gal-9 in H157 cells concurrently treated with KU-60019 (2 μmol/L or 4 μmol/L) and p-TKB1 inhibitor (GSK8612, 5 μmol/L) for indicated time. **J.** RT-qPCR analysis of IFNAR1 in vector control and IFNAR1-KD H157 cells. **K-M**. Immunoblot, RT-qPCR and ELISA analysis of Gal-9 in vector control and IFNAR1-KD H157 cells treated with KU-60019 (2 μmol/L or 4 μmol/L) or DMSO (vehicle control) for 5 days.** N-P.** Immunoblot, RT-qPCR and ELISA analysis of Gal-9 in H157 cells concurrently treated with KU-60019 (2 μmol/L or 4 μmol/L) and p-STAT1 inhibitor (Fludarabine, 0.25 μmol/L) for indicated time. n.s., not significant; *, P < 0.05; **, P < 0.01; ***, P < 0.001.

Based on the results above, we hypothesized that STING/IFNβ activation might contribute to ATMi-induced Gal-9 upregulation. To test the possibility, we pretreated the cells with H151, a specific STING inhibitor, and found that the induction of Gal-9 protein in response to ATM inhibition was completely suppressed in the presence of H151 (Fig. 4A). Similarly, the increase in Gal-9 mRNA and secretion was also abrogated upon H151 pretreatment in H157 and A549 cells, as determined by real-time PCR (Fig. 4B, Fig. S4A) and ELISA (Fig. 4C, Fig. S4B), concomitant with a decrease in IFNβ (Fig. S4C-E). To further validate the results, we generated STING stable knockdown H157 cells (Fig. 4D), and confirmed that STING depletion significantly prevented the increase in Gal-9 protein expression and secretion following ATM inhibition (Fig. 4E and F). In addition, inhibition of TBK1, a critical downstream kinase of STING, abrogated the induction of Gal-9 following ATM inhibition, as determined by levels of Gal-9 mRNA, protein and secretion (Fig. 4G-I). These results suggest that STING-TBK1 pathway is required for ATMi-induced Gal-9 upregulation. Moreover, knockdown of IFNAR1 (Fig. 4J), one of the two subunits of IFNβ receptor, resulted in a significant abrogation of increase in Gal-9 protein in response to ATM inhibition in H157 cells (Fig. 4K). Similarly, the upregulation of Gal-9 mRNA expression and secretion upon ATMi treatment was also prevented upon IFNAR1 depletion (Fig. 4L and M). Consistently, pharmacological inhibition of STAT1 led to a marked attenuation in the upregulation of Gal-9 mRNA, protein and secretion in response to ATM inhibition (Fig. 4N-P, Fig. S4F), indicating the importance of IFNβ signaling for Gal-9 induction by ATMi. Together, these results demonstrated that ATM inhibition enhances Gal-9 in a STING-TKB1-IFNβ dependent manner.

### Gal-9 blockade combined with ATM inhibition potently suppresses tumor growth in multiple syngeneic mouse models

**Figure 5:**
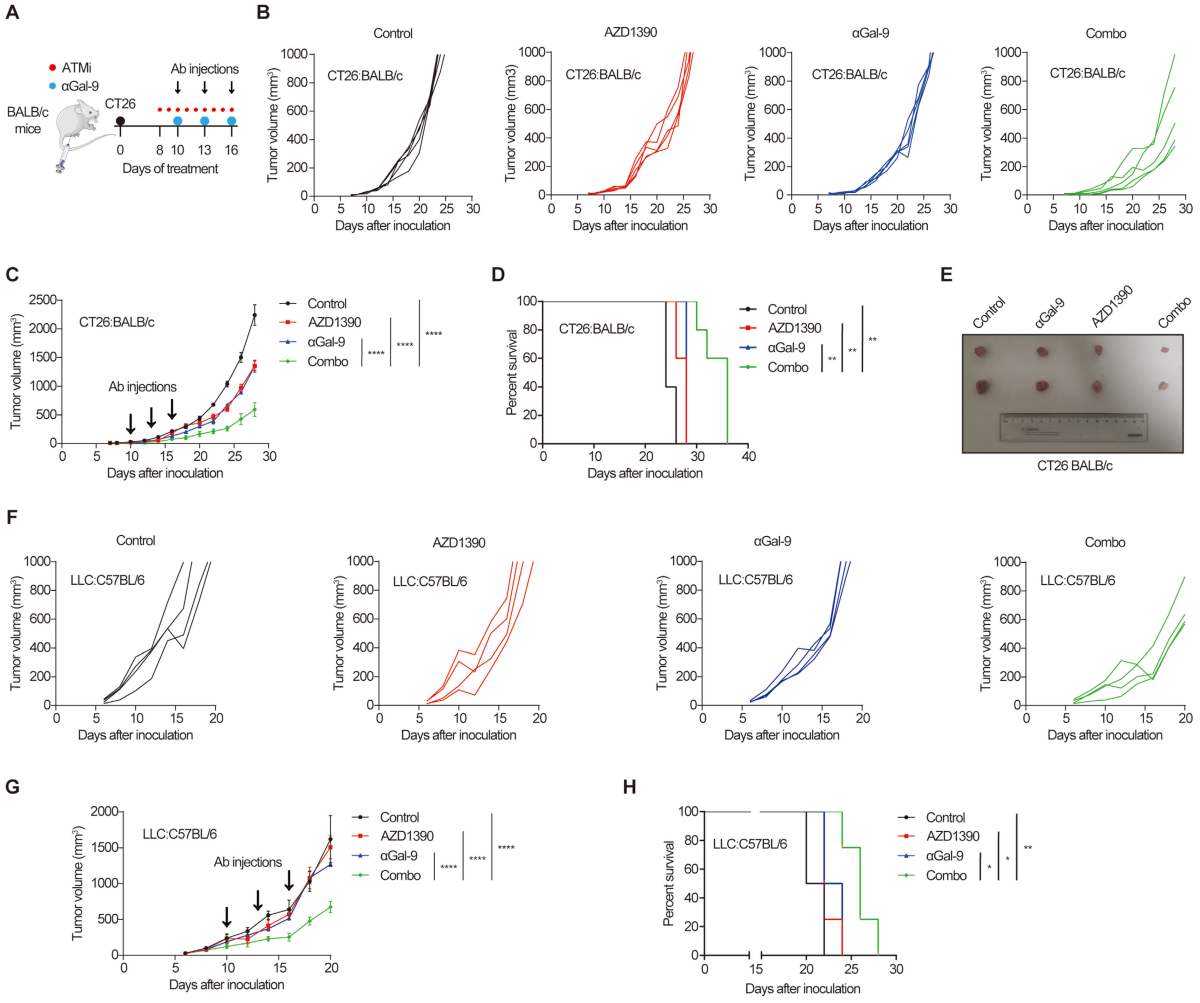
** Gal-9 blockade in combination with ATM inhibition potently suppresses tumor growth and prolongs mice survival. A.** A diagram of treatment strategy. BALB/c mice were inoculated with CT26 cells and then treated with isotype control, ATM inhibitor AZD1390 (5mg/kg for 9 times) alone, anti-Gal-9 antibody (100μg /dose for 3 times) alone, or their combination. **B.** Tumor growth curves of individual CT26-bearing BALB/c mice treated as indicated (n=5 mice/group). **C.** Average tumor growth of mice inoculated with CT26 tumor cells and subjected to the indicated treatments. Error bars represent SEM of the means. Anti-Gal-9 antibody treatment schedule is indicated by arrows. **D.** Survival curves of mice inoculated with CT26 tumors and subjected to the indicated treatments. **E.** The size of tumors harvested from individual CT26-bearing BALB/c mice on Day 14 after inoculation. **F.** Tumor growth curves of individual of LLC-bearing C57BL/6 mice treated with isotype control, ATM inhibitor AZD1390 (5mg/kg for 9 times) alone, anti-Gal-9 antibody (100μg /dose for 3 times) alone, or their combination (n=4 mice/group). **G.** Average tumor growth of mice inoculated with LLC tumor cells and subjected to the indicated treatments. Error bars represent SEM of the means. Anti-Gal-9 antibody treatment schedule is indicated by arrows. **H.** Survival curves of mice inoculated with LLC tumors and subjected to the indicated treatments. n.s., not significant; *, P < 0.05; **, P < 0.01; ***, P < 0.001.

The remarkable synergy of Gal-9 blockade and ATM depletion in tumor growth suppression (Fig. 1) prompted us to investigate the translational value of this observation by combining anti-Gal-9 therapy with AZD1390, a potent ATM inhibitor currently evaluated in clinical trials 27. As shown in Fig. 5A, BALB/c mice bearing CT26 tumors were treated with isotype control, ATM inhibitor AZD1390 (5 mg/kg for 9 times) alone, anti-Gal-9 antibody (100 μg/dose for 3 times) alone, or a combination of AZD1390 and anti-Gal-9 antibody. We found that while ATMi or anti-Gal-9 alone only moderately decreased tumor growth, their combination markedly improved tumor growth control and enhanced survival in CT26 syngeneic mouse model even with only three injections of anti-Gal-9 antibody (Fig. 5B-E). No significant body weight loss was observed in the combinational treatment group, suggesting the therapeutic doses used in for ATMi plus Gal-9 antibody were safe for animals (Fig. S5A). To confirm the efficacy of the combinational therapy, we used MC-38 murine colon tumor as an independent syngeneic model. Similarly, although the antitumor effects of anti-Gal-9 or ATMi alone were transient and minimal in MC-38 mouse model, their combination resulted in significantly greater inhibition of tumor growth than either single agent and prolonged overall survival (Fig. S5B-F). Next, we sought to assess whether ATM inhibition could enhance anti-Gal-9 therapy in the poorly immunogenic LLC murine lung cancer model 28 that is resistant to current immune checkpoint blockade 29. While AZD1390 or anti-Gal-9 alone had negligible tumor-suppressive effect in LLC tumors, their combination significantly enhanced the antitumor efficacy and extended the survival of mice (Fig. 5F-H). Therefore, our results from three murine tumor models strongly suggested that anti-Gal-9 therapy combined with ATM inhibition significantly reduces tumor growth and enhances the survival of host mice, even in poorly immunogenic tumor models that are refractory to current immune checkpoint therapy.

### Combination of anti-Gal-9 therapy with ATM inhibition increases T cell infiltration

**Figure 6:**
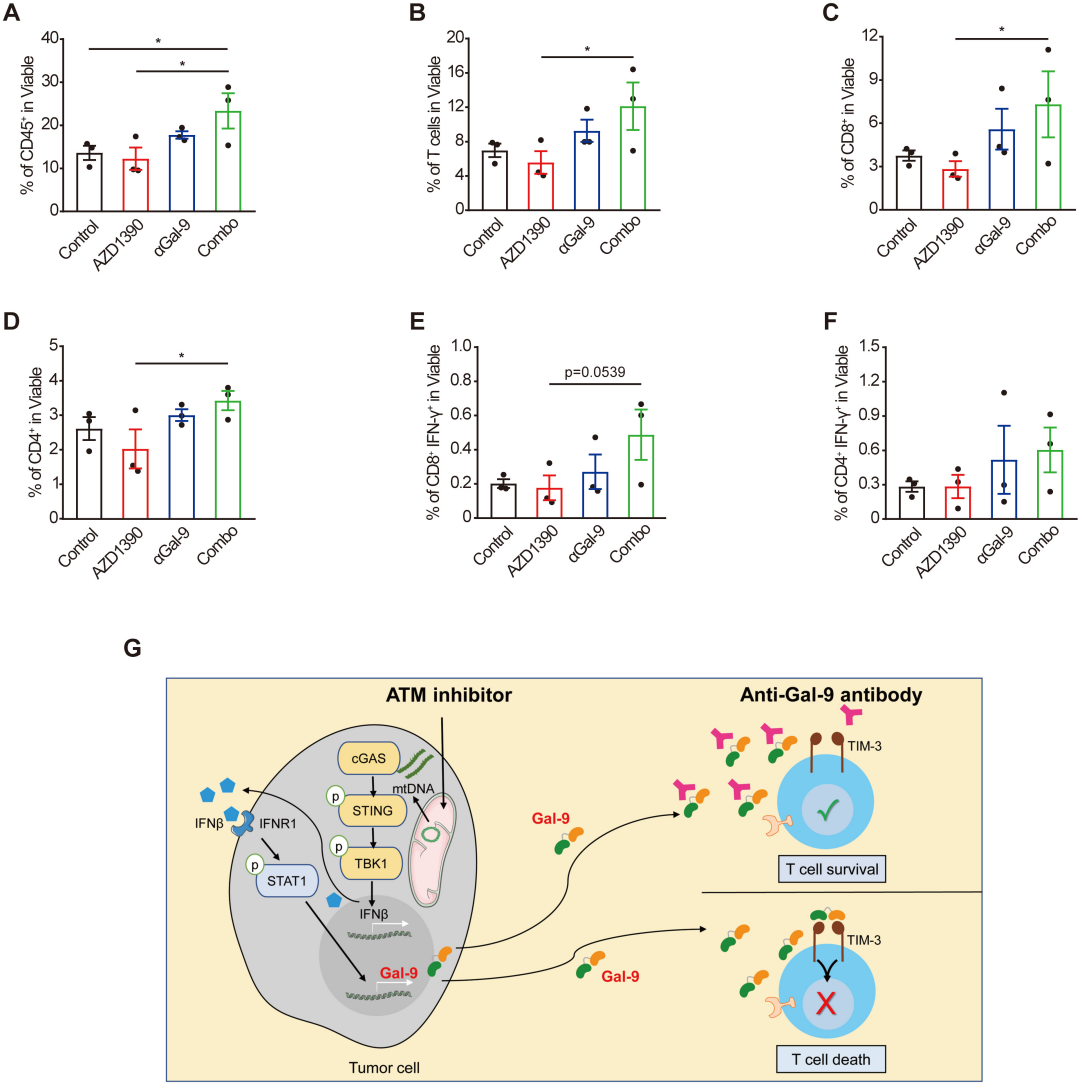
** Combination of Gal-9 blockade with ATM inhibition increases T cell infiltration.** Average frequency of tumor-infiltrating CD45+ cells **(A)**, total T cells **(B)**, CD8+T cells **(C)**, CD4+ T cells **(D)**, IFN-γ+CD8+ T cells **(E)** and IFN-γ+CD8+ T cells **(F)** in CT26 tumors harvested from BALB/c mice treated with isotype control, AZD1390 alone, anti-Gal-9 antibody (RG-1) alone, or their combination (n = 3 mice/group). Flow cytometric analysis was performed on day 14 after inoculation with CT26 tumor cells. Data represent the mean ± SEM. n.s., not significant; *, P < 0.05; **, P < 0.01; ***, P < 0.001.** (G)** Graphical summary of key findings. ATM inhibition triggers mitochondrial DNA (mtDNA) leakage and activates cGAS/STING/IFNβ innate immune pathway in tumor cells. Secreted IFNβ binds to IFNR1, activates the downstream STAT1 signaling and leads to Gal-9 induction. Gal-9 secreted from tumor cells interacts with TIM3 on T cells and induces T cell death. However, Gal-9 neutralizing antibody prevents Gal-9 from binding to TIM3 to increase T cell survival. Therefore, the combination of ATM inhibition and anti-Gal-9 increases T cell infiltration and induces anti-tumor immunity.

To investigate the alterations of tumor-infiltrating T cells upon the combination treatment that underlie the therapeutic efficacy, we performed flow cytometric analysis of CT26 tumor cells harvested from BALB/c mice on day14 after inoculation. The gating strategy for flow analysis is shown in Supplementary Fig.S6. Given that Gal-9 is known to bind to TIM-3 to induce T cell death, we posited that the Gal-9-neutralizing antibody is supposed to increase the numbers of T cells through preventing Gal-9 from binding to TIM3 to increase T cell survival 30. Indeed, anti-Gal-9 monotherapy caused a moderate increase in tumor infiltration of T cells, while ATMi alone did not have a detectable impact. In contrast, we observed a significant increase in the number of intratumoral CD45+ immune cells (Fig. 6A), total T-cells (Fig. 6B), CD8+ cytotoxic T-cell (Fig. 6C), and CD4+ T cells (Fig. 6D) in the combination treatment group, compared with single-agent ATMi or anti-Gal-9 treatment. Furthermore, we found increased percentage of IFN-γ positive CD8+ T (Fig. 6E) and CD4+ T cells (Fig. 6F) (although a statistical significance was not reached), suggesting that the combination treatment tended to promote T cell activation. These results indicate that the combination of anti-Gal-9 therapy and ATM inhibition synergistically increases T cell infiltration and thus promotes anti-tumor immunity.

## Discussion

Although anti-PD-1/PD-L1 immunotherapy has revolutionized the field of cancer treatment in multiple tumor types, only a small subset of patients benefits from such therapies, partially due to the “coldness” of the tumors, characterized by limited infiltration of lymphocytes (TILs) 31, particularly cytotoxic T cells. DDR-targeted therapies including ATM inhibitors have been shown to convert the “cold tumor” into “hot tumor” in some cancer types, through inducing cGAS/STING-mediated innate immunity. Consistent with this, herein, we show that inhibition of ATM induces the activation of cGAS-STING-IFNβ ubiquitously in multiple human and murine cancer cell lines. IFNβ plays a pivotal role in cancer immunosurveillance and anti-tumor immune response through activation of CD8+T cells, maturation of dendritic cells, and inhibition of regulatory T cells, etc. 32. However, increasing evidence suggests that persistent IFNβ production can lead to immunosuppressive effects that promote cancer 33,34. In this study we reveal Gal-9 upregulation via the STING-IFNβ pathway as a factor that limits the anti-tumor efficacy of ATM inhibition (Fig. 6G). Tumors highly expressing Gal-9 are thus likely to be T cell-inflamed “hot” tumors, which might underlie the positive correlation of Gal-9 with clinical outcome in some tumors 35. Although our previous study showed that Gal-9 could be upregulated by exogenous IFNβ in tumor cells 8, the role of STING signaling-induced endogenous IFNβ in Gal-9 regulation remains largely unknown. We demonstrate that Gal-9 expression can be increased by tumor cell-intrinsic IFNβ in response to ATM inhibition in a STING-dependent manner. Therefore, our and others' studies suggest that ATM inhibition acts as a double-edged sword to modulate immune response. On the one hand, it activates cGAS/STING to boost tumor immunogenicity, on the other hand, it induces the expression of immunosuppressive regulators, such as PD-L1 21 and Gal-9, to inhibit T cell activation and elicit T cell death, etc. Intriguingly, although we found that PD-L1 could also be upregulated in response to ATM inhibition, the extent of its alteration is minimal compared with that of Gal-9, suggesting that Gal-9, but not PD-L1, likely plays a predominant role in mediating the immunomodulation by ATMi. This study thus uncovers induction of tumoral Gal-9 as another important mechanism mediating immune escape that could be targeted for cancer immunotherapy. Our findings thus deepen the understanding of the immunological effects of DDR-targeted therapies and provide a rationale for a novel combinational modality to enhance the efficacy of the ATM inhibitors that are currently undergoing in a number of clinical trials 20,27.

Gal-9 is emerging as a promising therapeutic target for cancer immunotherapy recently. Anti-Gal-9 antibody in combination with an agonist antibody to the T cell co-stimulatory receptor GITR (glucocorticoid-induced tumor necrosis factor receptor-related protein) significantly outperforms monotherapy with either agent in suppressing tumor growth and improving survival in the mouse models of colon cancer and TNBC 8. In a pancreatic mouse model, the combination of Gal-9 neutralization and PD-1 blockade slows tumor progression and extends mouse survival 6. An anti-Gal-9 antibody, LYT-200, is currently evaluated in a clinical trial (NCT04666688), alone and in combination with chemotherapy or anti-PD-1 in patients with metastatic solid tumors 36. Therefore, Gal-9 neutralizing antibody has a great potential for cancer immunotherapy when combined with other modalities. Here, we demonstrate that anti-Gal-9 therapy in combination with ATM inhibition synergically suppresses tumor growth and prolongs survival in three syngeneic mouse models, including colon tumor and the poorly-immunogenic LLC lung tumor which generally lacks TILs and is unresponsive to anti-PD-1/PD-L1 therapy 37. These results envisage a novel anti-Gal-9-based combination strategy for a variety of human cancers, including those resistant to current anti-PD-1 immunotherapies. Based on our and other findings that ATM inhibition sensitize tumors to both anti-PD-1 21,22 and anti-Gal9 therapies, it is conceivable that triple combination of ATMi with anti-PD-1 and anti-Gal-9 would produce an even more potent anti-tumor effect. It will be intriguing to investigate these combination modalities in preclinical and clinical investigations in the future. Importantly, given that ATM has been shown to be essential for T cell and B cell maturation and function 38,39 it is conceivable that ATMi will have undesirable effect on immune cells, which should be taken into consideration for long-term treatment. This might partially explain why anti-Gal-9 showed lower efficacy in mice with ATMi treatment than those with depletion of tumoral ATM. As DNA damage in general primes the type I IFN system via the STING pathway 40, our work raises the exciting possibility that anti-Gal-9 plus other DDR-targeted therapies, such as PARP inhibition, also enhance antitumor immune responses leading to durable antitumor immunity.

Our study reveals some potential biomarkers that may predict the response to anti-Gal-9 treatment, such as the “hotness” of TME, and high levels of intratumoral Gal-9, which is induced by ATM deficiency. Germline mutations of ATM result in A-T syndrome, a cancer-predisposing disorder. ATM loss/mutation is commonly found in a range of sporadic cancers 19. For example, the deficiency of ATM protein evaluated by immunohistochemistry (IHC) assay is observed in over 40% of lung adenocarcinomas 41. Our findings thus are clinically important by revealing Gal-9 as a promising therapeutic target for A-T Syndrome and ATM-deficient tumors. Therefore, ATM loss/mutation represent as a predictive biomarker to stratified patients who would response to Gal-9 blockade. Moreover, as ATM inhibitors have already been extensively evaluated in clinical trials, our work underscores Gal-9-targeted cancer immunotherapy as a promising modality that can be evaluated in future clinical trials in combination with these ATM inhibitors.

Consistent with Gal-9 being a TIM-3 ligand that induces T cell death, we found that Gal-9 blockade led to an increase in the infiltration of T cells. Notably, the combination of anti-Gal-9 with ATMi synergistically induces significantly higher numbers of intratumoral T cells, compared with single-agent ATMi or anti-Gal-9 treatment. Intriguingly, we also found a trend of increase in the numbers of IFNγ-positive CD4+ and CD8+ cells in either anti-Gal-9 or the combination treatment group, suggesting that Gal-9 blockade may act to reinvigorate T cell activity in an unknown mechanism. Although TIM-3 is a key receptor that mediates Gal-9 function, other TIM-3-independent mechanisms of Gal-9 actions have also been reported, such as Treg expansion and differentiation 7,42, macrophage polarization 6 and natural killer cell function 43. Thus, given the pleotropic roles of Gal-9 in multiple immunomodulatory pathways, further experiments are warranted to investigate the impact of anti-Gal-9 therapy combined with ATM inhibition on other immune cells in the TME.

In summary, the study uncovers an intriguing link between DDR and an “immune checkpoint” protein Gal-9. Induction of Gal-9 via STING/IFNβ may represent a novel mechanism for tumors to invade immunosurveillance, thereby limiting the anti-tumor efficacy of DDR-targeted therapies. The findings have important clinical implications by highlighting Gal-9 as a promising therapeutic target for A-T Syndrome and ATM-deficient tumors and revealing combining ATM inhibition with anti-Gal-9 therapy as a novel strategy for cancer treatment. Our study thus provides a strong rationale for the use of ATM as both a predictive biomarker and a therapeutic target to enhance anti-Gal-9 therapy in future clinical trials. These findings will open a new line of immunotherapies for a wide variety of malignancies, including those with primary or adaptive resistance to PD-1/PD-L1 blockade.

## Supplementary Material

Supplementary figures and tables.
