# Supplementary material for: Galectin-9 blockade synergizes with ATM inhibition to induce potent anti-tumor immunity
Source: Int J Biol Sci. 2023 Jan 16;19(3):981–93. doi: 10.7150/ijbs.79852 (PMC9909994; doi:10.7150/ijbs.79852)
Supplement: Supplementary file 1 — Supplementary figures and tables. [file ijbsv19p0981s1.pdf]

Supporting information

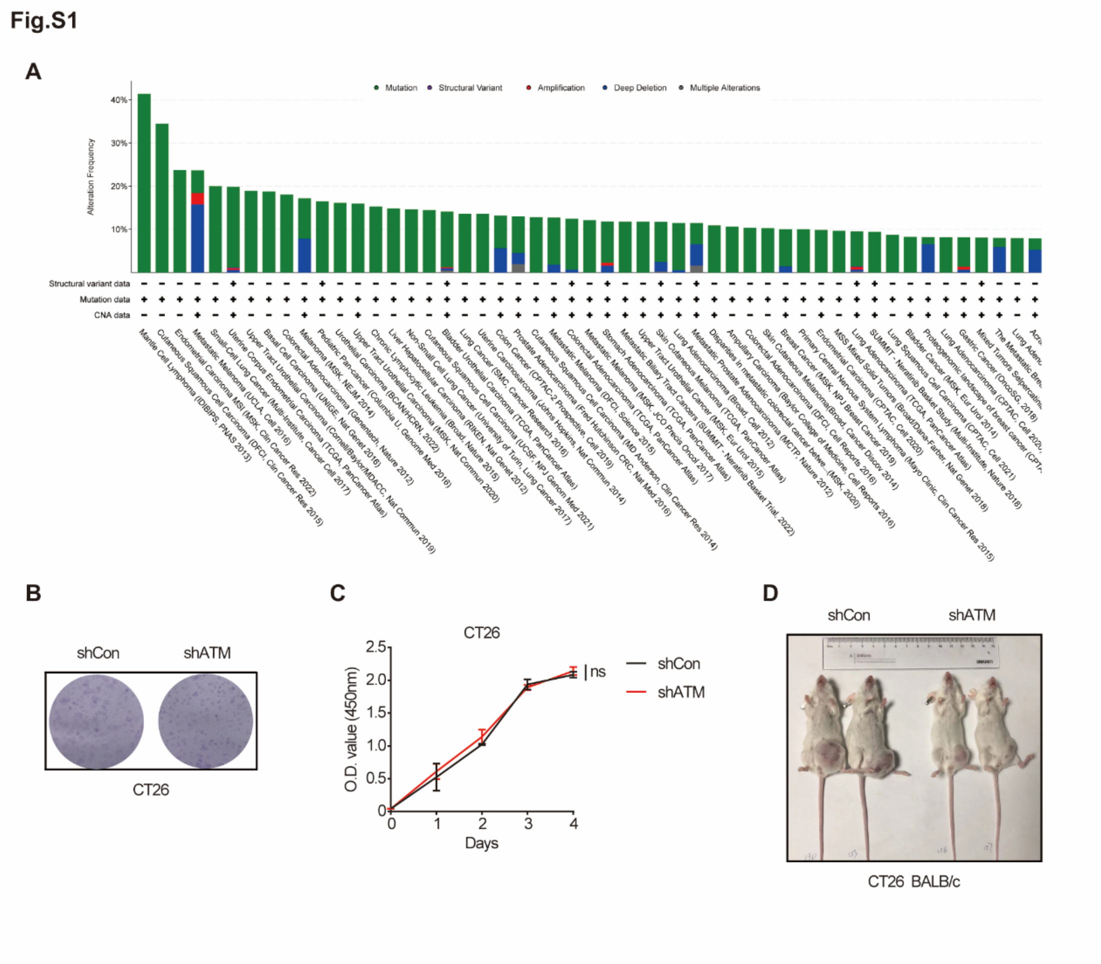

Fig.S1 ATM gene alterations in human cancers and the effect of ATM depletion on murine tumor growth in vitro and in vivo. A. The frequency of ATM gene alterations in various cancer types. ATM was queried against all entries in the curated non-redundant dataset on c-Bioportal, accessed November 2022. Green: mutation; purple: structural variant; red: amplification; blue: deep deletion; dark grey: multiple alterations. **B.** Growth evaluation of vector control or ATM-KO CT26 cells by crystal violet staining. **C.** Growth evaluation of vector control or ATM-KO CT26 cells by CCK-8 assay. **D.** The size of tumors of individual BALB/c mice inoculated with vector control or ATM-KO CT26 cells.

Fig.S2

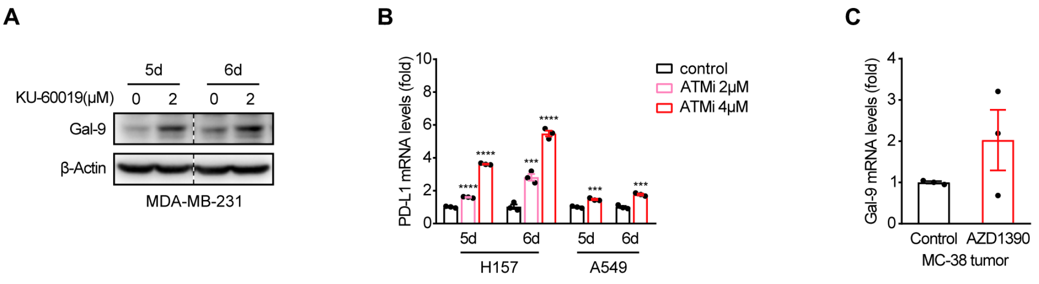

Fig.S2 The effect of ATM inhibition on Gal-9 or PD-L1 expression in tumor cells in vitro and in vivo. A. Immunoblot analysis of Gal-9 in MDA-MB-231 cells treated with KU-60019(2 μmol/L or 4 μmol/L) or DMSO (vehicle control) for indicated time. **B.** RT-qPCR analysis of PD-L1 mRNA levels in H157 and A549 cancer cells in response to KU-60019(2 μmol/L or 4 μmol/L) or DMSO (vehicle control) for indicated time. **C.** RT-qPCR analysis of Gal-9 in AZD1390-treated MC38 tumors harvested from C57BL/6J syngeneic mice. n.s., not significant; *, $P < 0.05$; **, $P < 0.01$; ***, $P < 0.001$.

Fig.S3

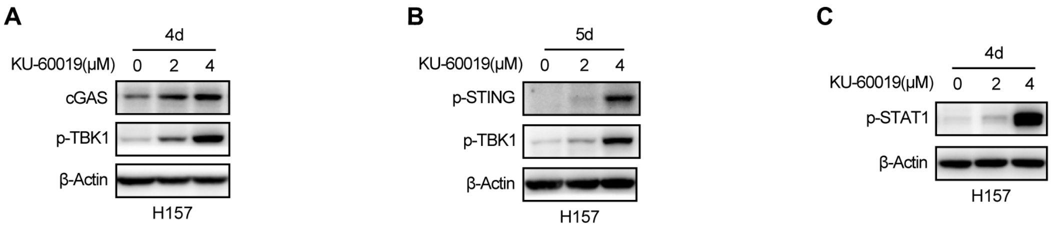

Fig.S3 ATM inhibition induces cGAS-STING-IFN β activation. A. Immunoblot analysis of cGAS, p-STING and p-TBK1 levels in H157 cells in response to KU-60019(2 μmol/L or 4 μmol/L) or DMSO (vehicle control) for 4 or 5 days. **B.** Immunoblot analysis of p-STAT1 in H157 cells in response to KU-60019(2 μmol/L or 4 μmol/L) or DMSO (vehicle control) for 4 days. n.s., not significant; *, $P < 0.05$; **, $P < 0.01$; ***, $P < 0.001$.

Fig.S4

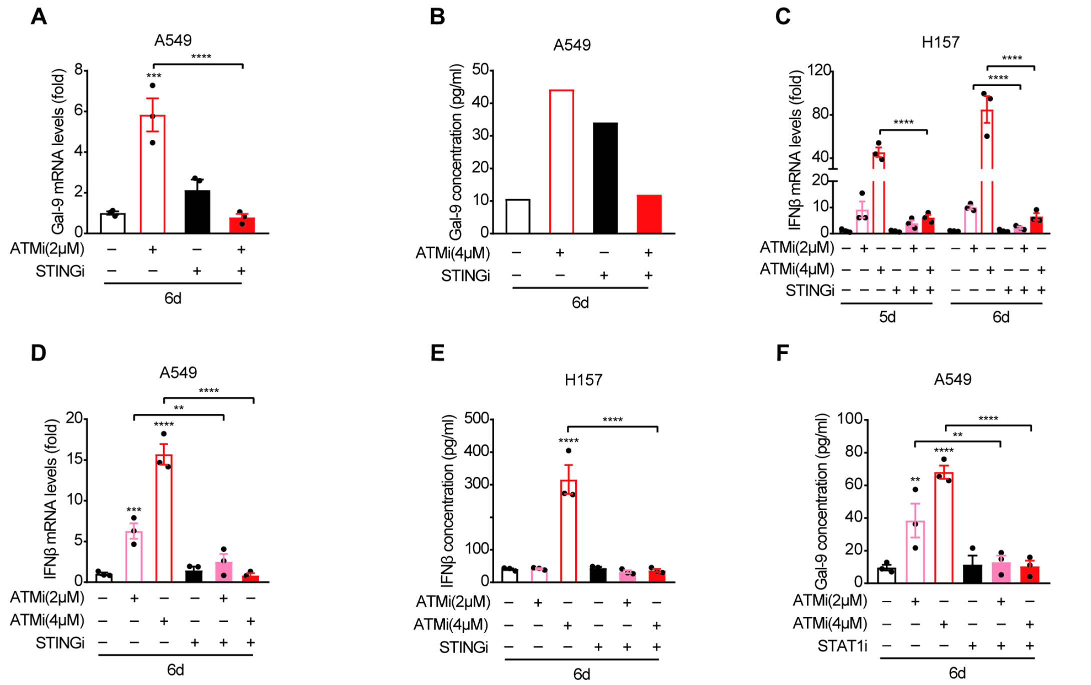

Fig.S4 STING or STAT1 inhibition abrogates Gal-9 induction in response to ATMi treatment. A. RT-qPCR analysis of Gal-9 in A549 cells concurrently treated with KU-60019(2 μmol/L or 4 μmol/L) and STING inhibitor (H151, 10 μmol/L) for indicated time. **B.** ELISA analysis of Gal-9 in A549 cells concurrently treated with KU-60019(2 μmol/L or 4 μmol/L) and STING inhibitor (H151, 10 μmol/L) for indicated time. **C and D.** RT-qPCR analysis of IFNβ in H157 and A549 cells concurrently treated with KU-60019(2 μmol/L or 4 μmol/L) and STING inhibitor (H151, 10 μmol/L) for indicated time. **E.** ELISA of IFNβ in H157 cells concurrently treated with KU-60019(2 μmol/L or 4 μmol/L) and STING inhibitor (H151, 10 μmol/L) for indicated time. **F.** ELISA analysis of Gal-9 in A549 cells concurrently treated with KU-60019(2 μmol/L or 4 μmol/L) and p-STAT inhibitor (fludarabine, 0.5 μmol/L) for indicated time. n.s., not significant; *, $P < 0.05$; **, $P < 0.01$; ***, $P < 0.001$.

Fig.S5

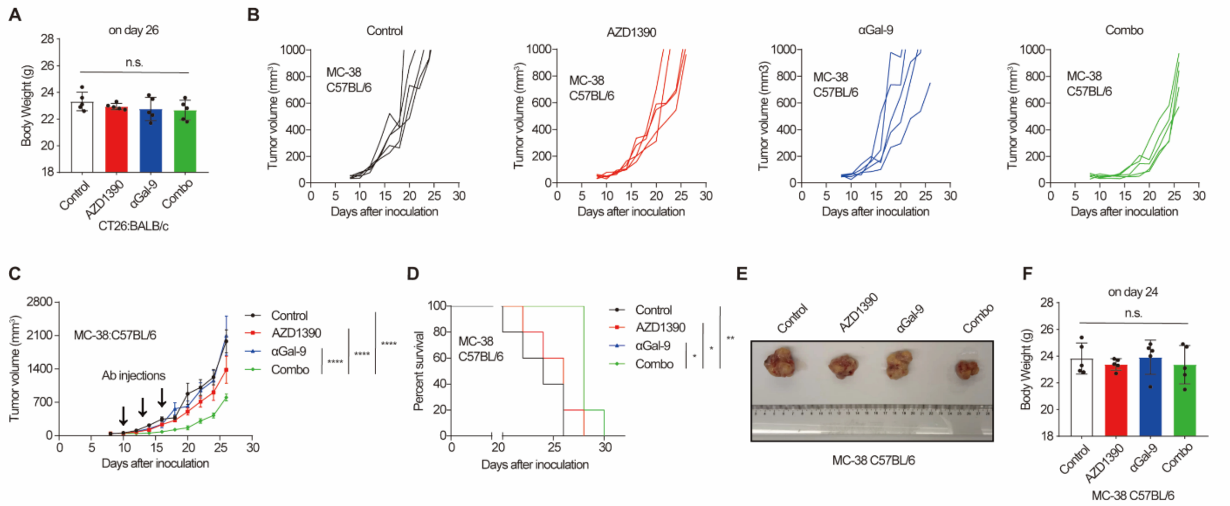

Fig.S5 Co-inhibition of anti-Gal-9 and ATM significantly reduces tumor growth in MC38 syngeneic mouse model. A. CT26-bearing BALB/c mice were treated as indicated and the body weight was measured on day 26 after tumor inoculation. **B**. Tumor growth curves of individual MC-38-bearing C57BL/6 mice treated with isotype control, then treated with isotype control, ATM inhibitor AZD1390 (5mg/kg for 9 times) alone, anti-Gal-9 antibody (100μg /dose for 3 times) alone, or their combination (n=5 mice/group). **C.** The average tumor growth of mice inoculated with MC-38 tumor cells and subjected to the indicated treatments. Error bars represent SEM of the means. Anti-Gal-9 antibody treatment schedule is indicated by arrows. **D.** Survival curves of mice inoculated with MC-38 tumors and subjected to the indicated treatments. **E.** The size of tumors harvested from individual MC38-bearing C57BL/6 mice treated as indicated after tumor inoculation. **F.** MC-38-bearing C57BL/6 were treated as indicated and the body weight was measured on day 24 after tumor inoculation. n.s., not significant; *, P < 0.05; **, P < 0.01; ***, P < 0.001.

Fig.S6

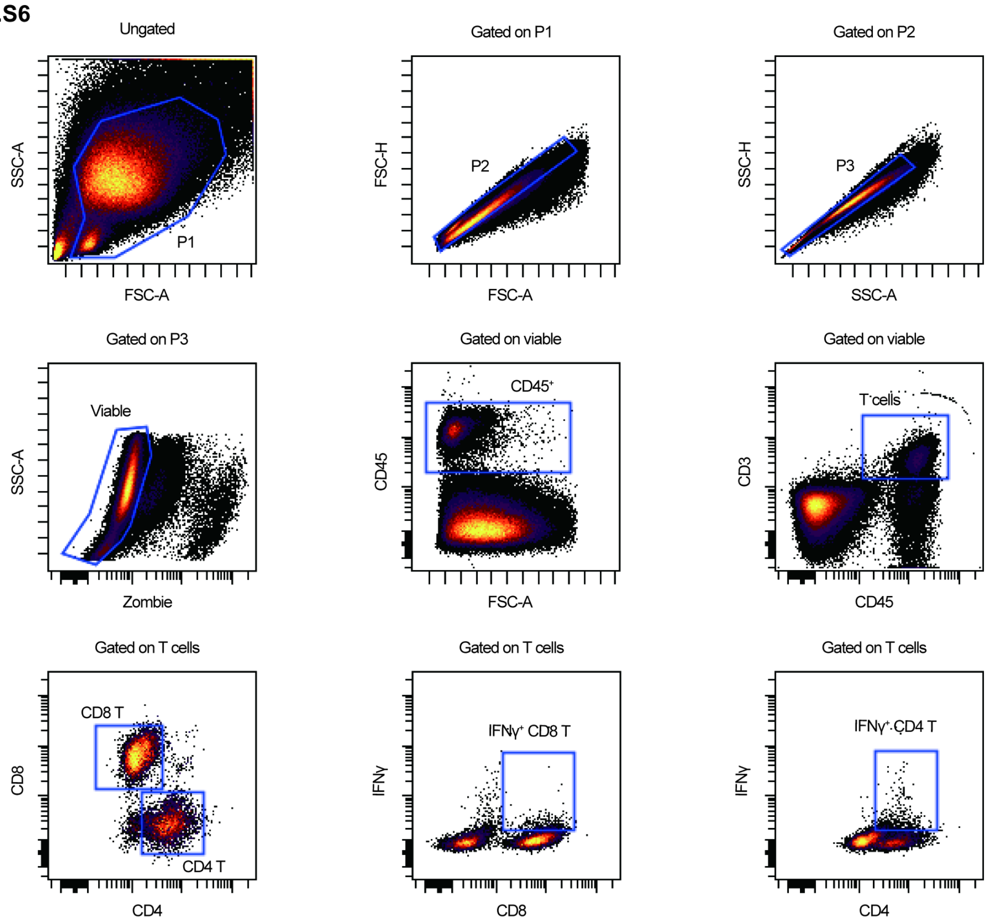

Fig.S6 Gating strategy for flow cytometric analysis of tumor-infiltrating T cells. CT26 tumors harvested from BALB/c mice were stained with indicated fluorescence-conjugated antibodies and analyzed by flow cytometry. Data were calculated using the cytobank website.

Supplementary Table S1. Primer sequences for qPCR

Target gene	Sequence
Human-GAPDH(F)	GGAGCGAGATCCCTCCAAAAT
Human-GAPDH(R)	GGCTGTTGTCATACTTCTCATGG
Human-galectin-9(F)	TCTGGGACTATTCAAGGAGGTC
Human-galectin-9(R)	CCATCTTCAAACCGAGGGTTG
Human-IFNβ(F)	GCTTGGATTCCTACAAAGAAGCA
Human-IFNβ(R)	ATAGATGGTCAATGCGGCGTC
Human-IFNAR1(F)	AACAGGAGCGATGAGTCTGTC
Human-IFNAR1(R)	TGCGAAATGGTGTAAATGAGTCA
Mouse-GAPDH(F)	AGGTCGGTGTGAACGGATTTG
Mouse-GAPDH(R)	TGTAGACCATGTAGTTGAGGTCA
Mouse-galectin-9(F)	TCAGTGCCCAGTCTCCATACA
Mouse-galectin-9(R)	CTCCTTGGATTGGTCCAGTAAAG
Mouse-IFNβ(F)	CAGCTCCAAGAAAGGACGAAC
Mouse-IFNβ(R)	GGCAGTGTAACTCTTCTGCAT
Mouse-ATM(F)	GATCTGCTCATTTGCTGCCG
Mouse-ATM(R)	GTGTGGTGGCTGATACATTTGAT

Supplementary Table S2. shRNA sequences for gene knockdown

Target gene	Sequence
Human-STING-sh1	CCGGATTCGAACTTACAAT
Human-STING-sh2	ATGGTCATATTACATCGGA
Human-STING-sh3	TGGCATGGTCATATTACAT
Human-IFNAR1	TTAGTGATTCATTCCATAT.
Mouse-ATM-sh1	TGAGACAAATAATGTCTTT
Mouse-ATM-sh2	GTGGAGATTTCTCAATCTT
Mouse-ATM-sh3	TACTAGAATACGGACATAT
